# Elucidating the genotoxicity of *Fusobacterium nucleatum*-secreted mutagens in colorectal cancer carcinogenesis

**DOI:** 10.1186/s13099-024-00640-w

**Published:** 2024-09-27

**Authors:** Wenye Xu, Yuchen Zhang, Dongjiao Chen, Dan Huang, Yang Zhao, Wei Hu, Ling Lin, Yingzhi Liu, Shilan Wang, Judeng Zeng, Chuan Xie, Hung Chan, Qing Li, Huarong Chen, Xiaodong Liu, Sunny H. Wong, Jun Yu, Francis K. L. Chan, Matthew T. V. Chan, Siew C. Ng, William K. K. Wu, Lin Zhang

**Affiliations:** 1 Microbiota I-Center (MagIC), Hong Kong, SAR China; 2 Department of Anesthesia and Intensive Care and Peter Hung Pain Research Institute, Faculty of Medicine, The Chinese University of Hong Kong, Hong Kong, SAR China; 3 Obstetrics Department, The Third Affiliated Hospital of Zhengzhou University, Zhengzhou, China; 4 Department of Pharmacology, Shenzhen People’s Hospital, (The Second Clinical Medical College, Jinan University, The First Affiliated Hospital, Southern University of Science and Technology), Shenzhen, 518020 China; 5 Department of Gastroenterology, Shenzhen Hospital, Southern Medical University, Shenzhen, Guangdong China; 6 Department of Medicine and Therapeutics, Faculty of Medicine, The Chinese University of Hong Kong, Hong Kong, SAR China; 7 State Key Laboratory of Digestive Diseases, Institute of Digestive Disease, Li Ka Shing Institute of Health Sciences, The Chinese University of Hong Kong, Hong Kong, SAR China; 8 CUHK Shenzhen Research Institute, Shenzhen, 518172 China; 9 Department of Gastroenterology, The First Affiliated Hospital of Nanchang University, Nanchang, 330006 Jiangxi China; 10 Lee Kong Chian School of Medicine, Nanyang Technological University, Singapore, 639798 Singapore; 11 Centre for Gut Microbiota Research, The Chinese University of Hong Kong, Hong Kong, SAR China

**Keywords:** *F. nucleatum*, CRC, DL-homocystine, Allantoic acid, DNA damage

## Abstract

**Background:**

*Fusobacterium nucleatum* (*F. nucleatum*) is one of the key tumorigenic bacteria in colorectal cancer (CRC), yet how *F. nucleatum* is involved in colorectal cancer carcinogenesis remains unknown.

**Results:**

In the present study, we carried out PathSeq analysis on RNA sequencing data from the 430 primary colon adenocarcinomas in TCGA database to assess the relationship between patients’ survival and *F. nucleatum* abundance. Among patients with cecum and ascending colon tumors, we found that *F. nucleatum* transcriptome abundance is positively correlated with mutation load. We further demonstrated that patients with both high tumoral abundance of *F. nucleatum* and high mutation load exhibited poorer survival and DNA damage. We furthermore determined that *F. nucleatum*-conditioned medium (Fn. CM) induces DNA damage in both in vitro and in vivo studies. In addition, two *F. nucleatum*-secreted mutagens, namely DL-homocystine and allantoic acid, were identified to lead to DNA damage.

**Conclusions:**

Our finding delineates the genotoxicity of *F.nucleatum*-secreted mutagens, which provides a basis for further work to investigate the role of *F. nucleatum* in the pathogenicity of CRC.

**Supplementary Information:**

The online version contains supplementary material available at 10.1186/s13099-024-00640-w.

## Introduction

The cancer-associated microbiota is known to influence many types of cancer development and progression [1–5], most notably in colorectal cancer (CRC) [6–10]. Unbiased genomic analyses have revealed an enrichment of *F. nucleatum* in human colon cancers and adenomas relative to non-cancerous colon tissues [11–13]. Stage-specific analyses showed that the relative abundance of *F. nucleatum* exhibited a progressive increase from early to late stages of carcinogenesis [11, 13]. These observations have been confirmed in studies of multiple CRC patient cohorts from different parts of the world [13–17]. Although *F. nucleatum* was first recognized as a passenger bacterium [18], emerging experimental findings supported a mechanistic role of *F. nucleatum* in driving tumorigenesis rather than acting as a microbial ‘passenger’ in CRC [19, 20]. Suggested the mechanisms included enhanced tumor cell metabolism, proliferation and metastasis [21–24], induced inflammation and suppressed the host immunity [25, 26], activated autophagy to confer resistance to chemotherapy [27] and reduced the efficiency of chemotherapy via the Toll-like receptor 4 (TLR4) pathway [28].

In the past few years, the understanding of the interplay between bacterial infection and genomic DNA damage has greatly increased [29–35]. Recent studies have demonstrated that pro-tumorigenic gut bacteria are associated with CRC development, and it has been postulated that some of these bacteria could contribute to enhanced mutagenesis [11, 12, 30]. Bacterial genotoxins could increase genome instability, result in DNA adducts [29, 35] and affect the host histone modification [36], which is critical to DNA repair after the DNA damage, ultimately promoting tumorigenesis. A well-known example is colibactin, a potent genotoxin that has captured the attention of both biologists and chemists due to its significant effect on human health and its potentially causative role in CRC initiation and progression [31, 32, 34]. New studies demonstrated a direct link between exposure of human intestinal epithelial cells to colibactin and two unique mutational signatures, single-base substitutions (SBS) and deletion [37, 38]. Furthermore, a significant step further by utilizing organoids to observe transformation has shown that cells exposed to colibactin-producing *Escherichia coli* (*E. coli*) for three hours could proliferate independently of the Wnt signal, a precursor to cancer and the mechanism is related to chromosomal restructuring [39].

Epidemiological studies demonstrated that *F. nucleatum*-high colonic lesions (either malignant or pre-malignant) bear certain mutations (BRAF, kirsten rat sarcoma virus (KARS), tumor protein p53 (TP53) and others) [16]. FadA, a novel adhesin of *F. nucleatum*, is shown to promote DNA damage and progression of *F. nucleatum*-induced CRC [40], indicating that *F. nucleatum* may promote genome instability and mutation. Chromosomal instability (CIN) is a major type of genomic instability, in which either the whole or parts of chromosomes are duplicated or deleted, leading to a range of karyotypic abnormalities and playing a complex role in cancer progression [41, 42]. One main form of CIN, the recurrent missegregation of whole chromosomes during cell division, leads to aneuploidy, a hallmark of most solid tumors [43], and a high level of CIN phenotype showed significantly poorer survival than the low in CRC [44]. These data and researched mice experiments support that there are links between *F. nucleatum* and chromosomal instability [45]. Although scientists have long reported that *F. nucleatum* is involved in CRC development, underlying mechanisms of how *F. nucleatum* leads to CRC initiation and development remained unknown as none of the mutagens or genotoxins has been identified from *F. nucleatum*.

With the completion of The Cancer Genome Atlas (TCGA) program, the amount of sequencing and expression data of different cancer types is gradually increasing. Our previous work in metagenomic profiling in CRC revealed an interacting network of oral pathogens [46, 47], which could serve as non-invasive markers for the early detection of CRC [48]. Pertinent to clinical practice, faecal microbe *F. nucleatum* is a non-invasive marker for colorectal adenoma and cancer [49]. In this study, we combined the TCGA dataset analysis, untargeted metabolites, and experiments in vitro and in vivo to analyze and identify the components of DNA damage secreted by *F. nucleatum*.

## Results

### F. nucleatum abundance in CRC tumor is correlated with poor survival and DNA damage

**Fig. 1:**
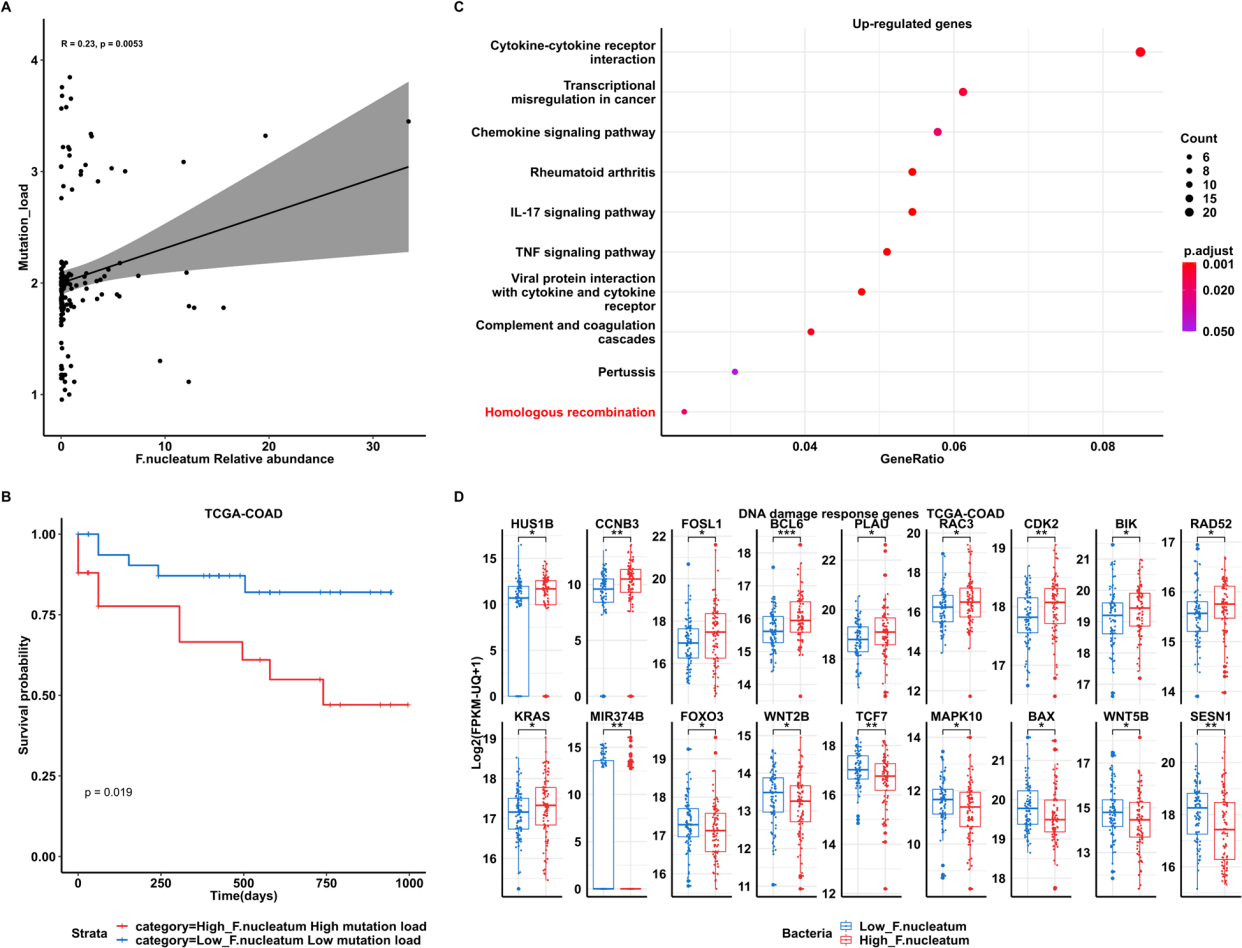
*F. nucleatum* abundance in CRC tumors is correlated with poor survival and DNA damage **A** Spearman’s correlation of *F. nucleatum* relative abundance (RA) with mutation load (log10 number of non-synonymous mutation per sample) in TCGA colon adenocarcinomas (COAD). **B** Kaplan–Meier curves of patient’s overall survival from TCGA-COAD based on *F. nucleatum* load (determined by PathSeq analysis) and mutation load. *P* values were determined by the Log-rank Mantel-Cox text. High *F. nucleatum* load: *F. nucleatum* RA values (> 50 percentile); low *F. nucleatum* RA values (< 50 percentile). High mutation load: log10 number of non-synonymous mutation per sample > 2: low mutation load: log10 number of non-synonymous mutation per sample < 2. **C** High *F. nucleatum* RA-associated genes enriched in DNA damage-related pathways. **D** Differential gene expression in High- and Low *F. nucleatum* samples

A total of 430 primary colon adenocarcinoma samples in TCGA cohort were included in the PathSeq analysis. We first explored the relationship between the abundance of bacteria and mutation load (Supplementary Fig. 1). *F. nucleatum* transcriptome abundance is positively correlated with mutation load (log10 number of non-synonymous somatic mutation per samples, Spearman’s test, R = 0.25, p = 0.0051) (Fig. 1A). Among the identified bacteria, *F. nucleatum* ranked among the top five in correlation with mutation load (Supplementary Fig. 1). Patients with both high tumoral abundance of *Fusobacterium* and high mutation load exhibited poorer survival (*Fusobacterium* relative abundance > 50th & mutation load > 2, *p* = 0.015) (Fig. 1B). Characteristics of colorectal cancer patients with high or low *F. nucleatum* relative abundance were summarized in Supplementary Table 1, indicating that patients with high *F. nucleatum* abundance are more likely to exhibit microsatellite instability (MSI), CpG island methylation abnormalities (CIMP-high and CIMP-low), as well as CMS1 (CIMP-high and MSI) and CMS2 (high somatic copy number alterations) phenotypes. Notably, three patients exhibited an abundance exceeding 10% in *F. nucleatum*, coupled with a mutation rate surpassing 2.5. The characteristics of these patients were summarized in Supplementary Table 2. To mitigate the impact of these outliers, we conducted a sensitivity analysis by excluding these three patients. The remaining data still exhibited a significant positive correlation between *F. nucleatum* abundance and mutation load (Spearman’s Rho = 0.17, p-value = 0.03). Additionally, the survival rate was notably lower for patients with higher levels of *F. nucleatum* and mutation load (Log-rank Mantel-Cox test, p-value = 0.02) (Supplementary Fig. 2), indicating a potential impact of *F. nucleatum* on the host's DNA instability. There are total 3571 genes that are differentially expressed between *F. nucleatum*-high (above the median) and *F. nucleatum*-low groups (below the median) (Wilcoxon test, *p* < 0.05). By KEGG pathway analysis (Fig. 1C), high *F. nucleatum* relative abundance-associated genes enriched in DNA damage-related pathways (homologous recombination). A distinct gene expression profile was identified in colorectal cancer samples with high levels of *Fusobacterium nucleatum*. Specifically, we observed an upregulation of 11 genes, namely *HUS1B, CCNB3, FOSL1, BCL6, PLAU, RAC3, CDK2, BIK, RAD52, KRAS*, and *MIR374B*. These genes are known to be involved in the DNA damage response or are recognized as oncogenes, highlighting their potential role in tumorigenesis [50–60].

Conversely, we found that 7 genes, including *FOXO3, WNT2B, TCF7, MAPK10, BAX, WNT5B,* and *SESN1*, were downregulated in samples with high levels of Fusobacterium nucleatum. Intriguingly, these genes are reported to have functions related to DNA damage repair [61–67], suggesting that their downregulation could impair the cell’s ability to repair DNA damage, thereby contributing to the progression of the disease (Fig. 1D). Taken together, our results showed that *F. nucleatum* in primary CRC tissues is associated with DNA damage and patients’ survival.

### *F. nucleatum induces DNA damage *in vitro* and *in vivo

**Fig. 2:**
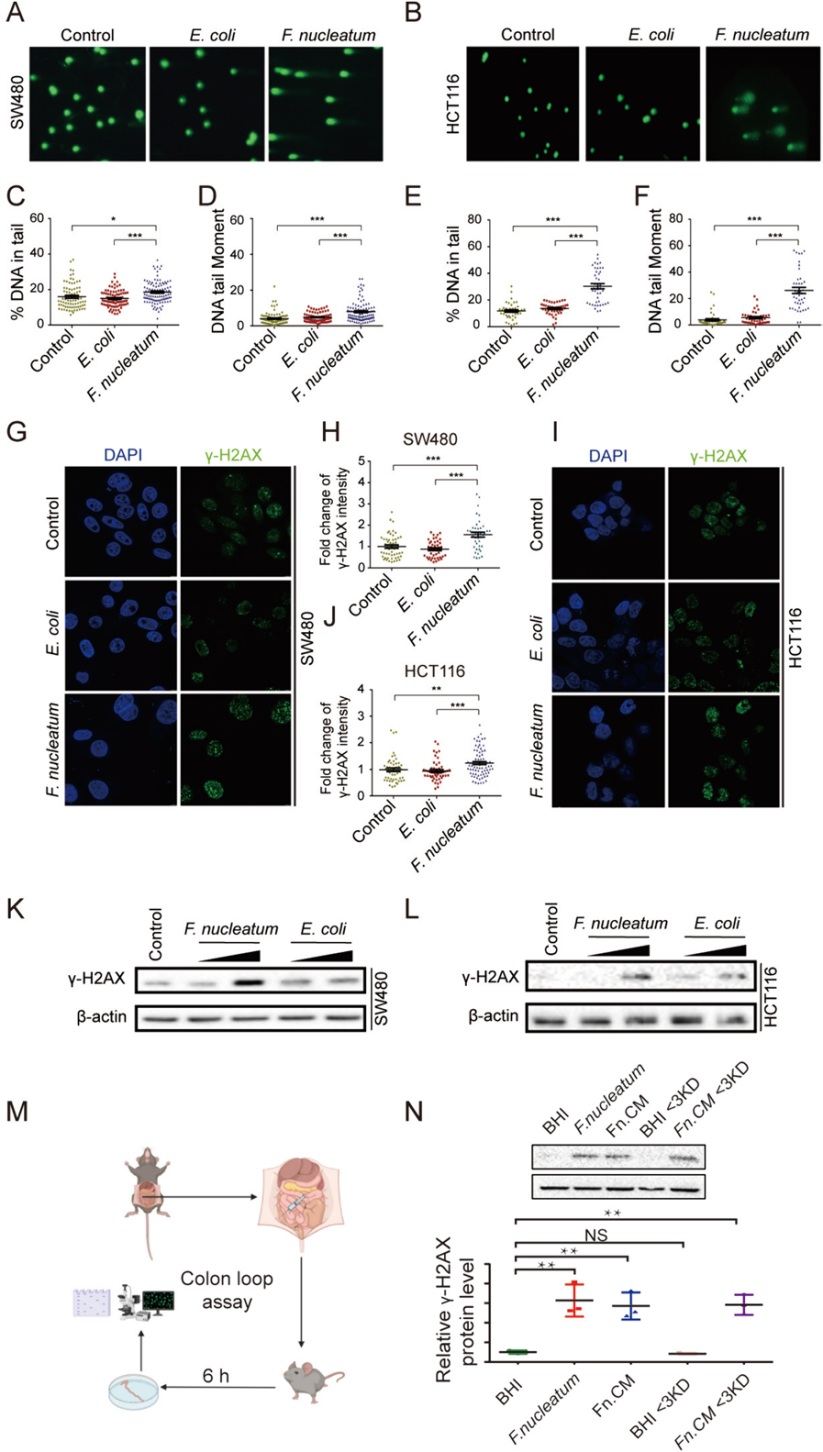
Exposure to *F. nucleatum* induces host DNA DSBs. Colon cancer cell lines SW480 and HCT116 were exposed to *F. nucleatum* (MOI = 50) or *E. coli* (MOI = 100) for 4 h per day under anaerobic conditions for 3 consecutive days. Cell lines SW480 and HCT116 were then harvested and subjected in neutral comet assay **A**, **B** and DSBs were quantified by DNA in tail **C**, **E** and DNA tail moment **D**, **F** γ-H2AX immunofluorescence stain was visualized by the confocal images of γ-H2AX (green) and DNA (blue) in cell line SW480 **G** and cell line HCT116 (**I**). **H**, **J** Fold change of γ-H2AX were compared by Wilcoxon rank-sum test. **K**, **L** γ-H2AX protein level was performed by Western blots. **M** C57BL/6 mice were anesthetized by intraperitoneal administration of ketamine and xylazine and their abdomen were disinfected with ethanol immediately before surgery. A midline laparotomy was performed, and a ligation was performed under the caecum and the other ligation was performed around 3 cm away from the first ligation. BHI, *F. nucleatum*, Fn.CM and metabolites were injected into the colon loop. After inoculation, the incision was closed. Mice were euthanized 6 h after surgery. The fraction of colon between two ligations were collected for DNA damage assay. **N** DNA damages were assayed by Western Blot. ***p ≤ 0.001; **p ≤ 0.01; *p < 0.05; NS, not significant. Results of A-L were derived from three independent experiments

**Fig. 3:**
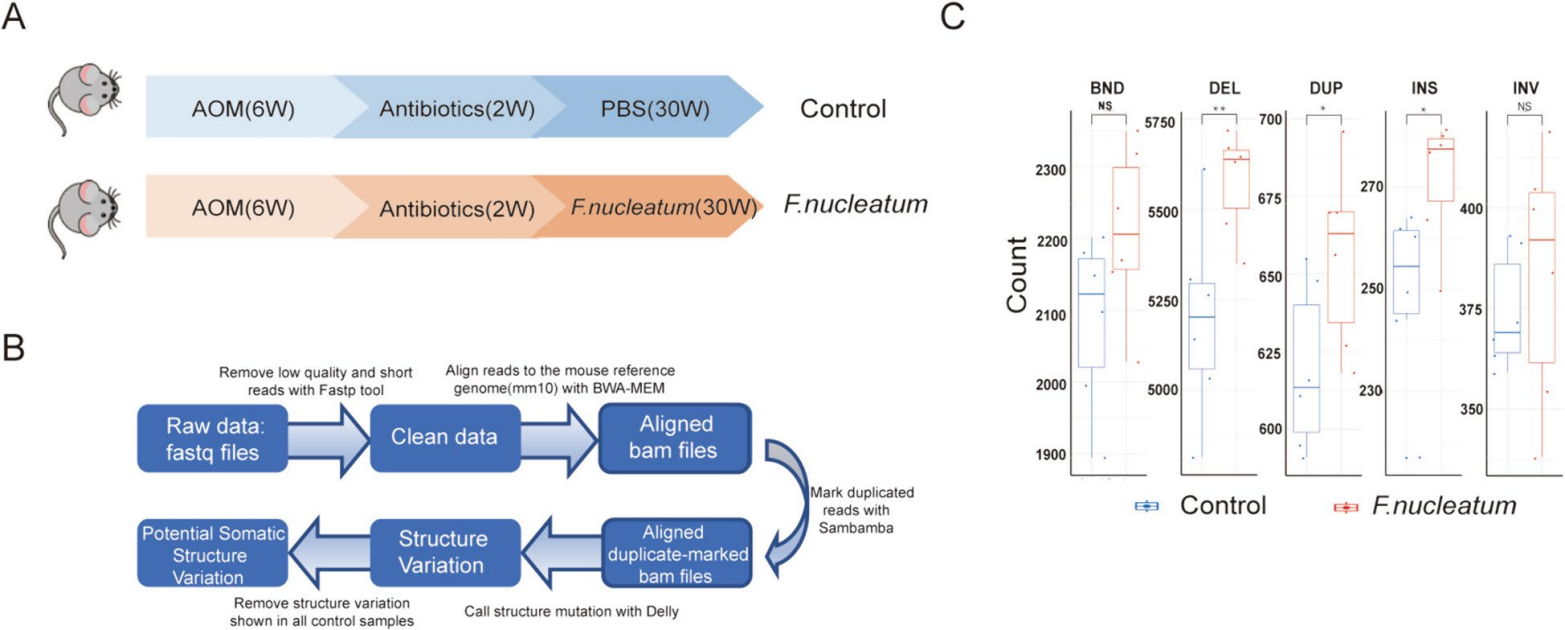
*F. nucleatum* induces DNA damage in vivo. C57BL/6 mice were administered with antibiotics through drinking water for 2 weeks. Then mice were administered with 10^8^ CFU *F. nucleatum* or PBS 5 times per week for 30 weeks by gavage. **A** DNA of colon tissue was extracted and performed whole genome sequence. **B** Pipeline of whole genome sequencing analysis. **C** Comparison of the number of different structure variations between *F. nucleatum* samples and control samples. BND: break-end; *DEL* deletion, *DUP* Duplication, *INS* Insertion, *INV* Inversion. ***p ≤ 0.001; **p ≤ 0.01; *p < 0.05; NS, not significant; Wilcoxon rank-sum test

We then investigated whether *F. nucleatum* could induce DNA damage by comet assay in vitro, which is a useful technique in the detection of DNA damage, particularly DNA strand breaks [43] (Fig. 2A, B). Colonic epithelial cells co-cultured with *F. nucleatum* showed a significant increase in the % of DNA tail (Fig. 2C, E) and DNA tail moment (Fig. 2D, F) compared with those without bacteria co-incubation or co-cultured with the non-pathogenic strain of *E. coli* as controls. Quantification of integrated intensities from single cells or Western blots with whole-cell lysate showed that *F. nucleatum* infection caused an elevation of γ-H2AX immunofluorescence intensity, foci number per cell (Fig. 2G–J), and protein levels (Fig. 2K, L, Supplementary Fig. 3). Further, we adopted a mouse intestinal loop model [29] to assess whether *F. nucleatum* could induce DNA damage in vivo. Colon loops were infected with *F. nucleatum* (1 × 10^9^ CFU/per 300 µl) or brain heart infusion (BHI) broth as control for 6 h (Fig. 2 M), separately. Western blot analyses of colonocytes indicated increased γ-H2AX in the intestinal loops infected with *F. nucleatum* as compared with BHI (Fig. 2N). To substantiate these results, a second in vivo mouse experiment was then conducted to evaluate the mutagenic role of *F. nucleatum* on CRC initiation. In the mouse chronic infection model, 6-week-old C57BL/6 male mice infected with *F. nucleatum* by oral administration for 30 weeks showed a significant DNA damaging effect than the BHI control group (Fig. 3A). During the experiment, the abundance of *F. nucleatum* increased following oral gavage from the 8th week onwards. Notably, at the 20th and 30th weeks, the *F. nucleatum* group exhibited a significant elevation in abundance compared to the control group (Supplementary Fig. 4). We then set out whole genome sequencing to explore the potential specificity of *F. nucleatum*-induced DNA damage by determining the characteristics of somatic structural variation in the mouse colon tissue (Fig. 3B). It is interesting that we identified signatures of somatic alterations consequential to double-strain breaks, including insertion, deletion, and inversion, in mouse colon tissues with *F. nucleatum* infection but not that from the BHI group (Fig. 3C). These results indicate that *F. nucleatum* could induce DNA damage in colonic epithelial cells, in the colon loop model and in mice.

### *F. nucleatum-conditioned medium (Fn. CM) induces DNA damage *in vitro* and *in vivo

**Fig. 4:**
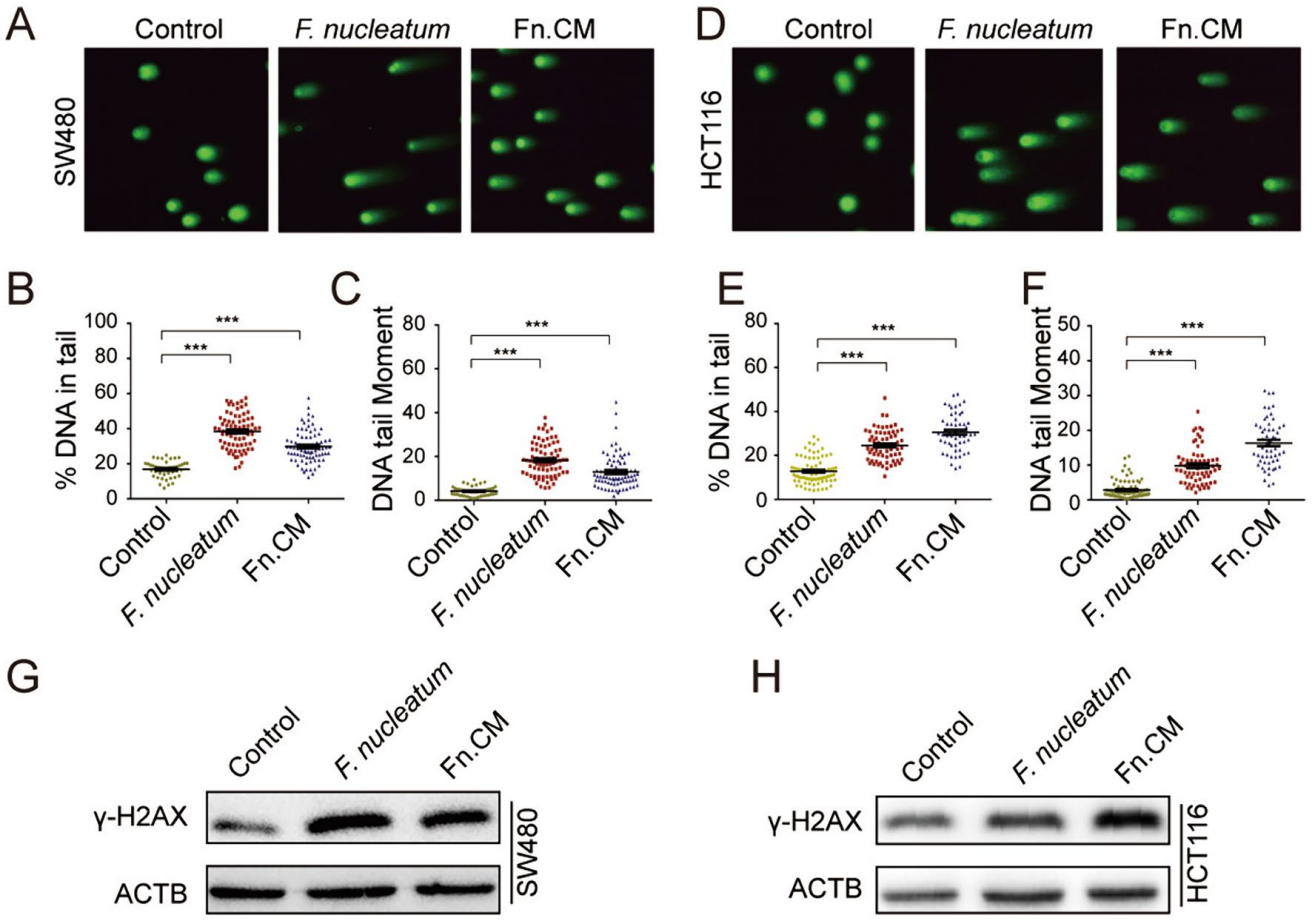
*F. nucleatum*-conditioned medium (Fn. CM) induces DNA damage in vitro. Colon cell lines SW480 and HCT116 were exposed to *F. nucleatum* or Fn. CM for 3 consecutive days. DNA damage was assayed by neutral comet assay **A**, **D** and quantified as DNA in tail **B**, **E** and DNA tail moment (**C**, **F**). **G**, **H** Protein levels of γ-H2AX were assessed by Western blots. ***p ≤ 0.001. Results were derived from three independent experiments

To understand more precisely how *F. nucleatum* induces DNA damage, we next explored whether such an effect could be attributed to *F. nucleatum* itself or its secreted products. Fn. CM was obtained by removing *F. nucleatum* with a 0.22 μm syringe filter and subsequently exposed to CRC cells. Comet assay and Western blots for γ-H2AX indicated that CM of *F. nucleatum* (Fn. CM) but not the control medium induced DNA damage (Fig. 4A–H). To test whether Fn. CM exhibits DNA damage in vivo, we used a mouse colon loops model. Again, Western blot results showed elevated γ-H2AX in the intestinal loops in Fn. CM group than that from BHI control group (Fig. 2N), suggesting that the DNA damaging effect of *F. nucleatum* was mainly mediated by the bacteria-secreted molecules.

### Identification of the F. nucleatum-secreted mutagen in Fn. CM

**Fig. 5:**
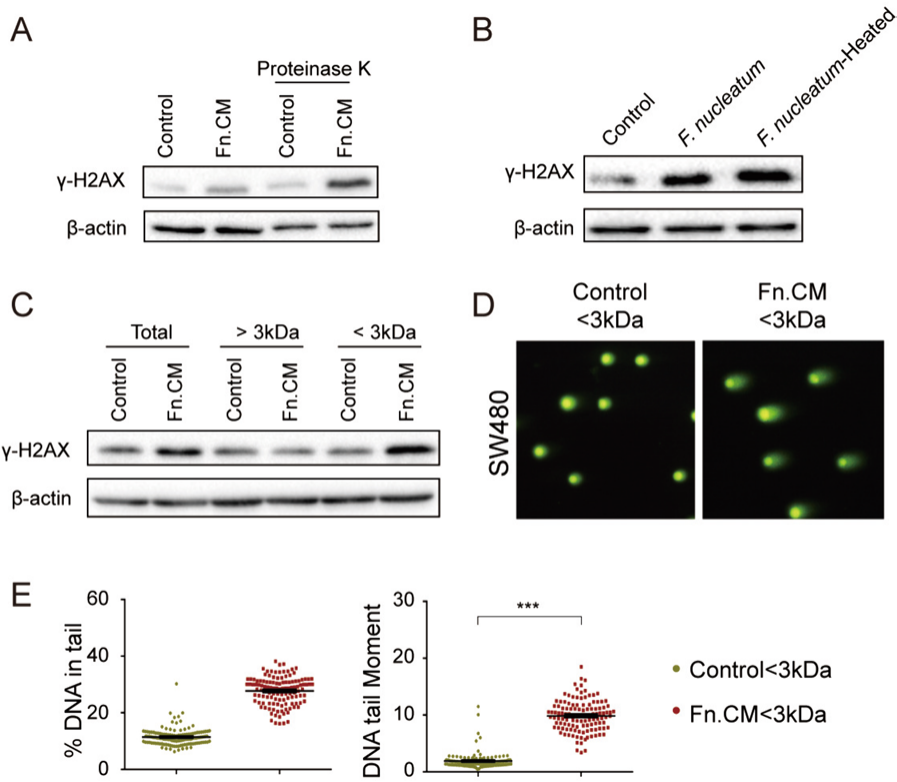
Isolation of the *F. nucleatum*-secreted mutagen-containing fractions in Fn. CM. Fn.CM was subject to digestion by protease K (50 μg/mL) **A** or heat inactivation (100 °C for 30 min) **B** and co-cultured with SW480 cell line. Protein levels of γ-H2AX were detected by Western blots. Fn. CM was separated using 3-kDa molecular weight cutoff (MWCO) membranes and the DNA-damaging effect was assessed by Western blots **C** and comet assay (**D**, **E**). ***p ≤ 0.001. Results were derived from three independent experiments

To determine the characteristics of the mutagen(s), the DNA-damaging effect of the digested or heat-inactivated Fn.CM was then assessed by comet assay and γ-H2AX protein expression in both SW480 and HCT116 cells. As shown in Fig. 5, DNA damage was still increased in cells exposed to Fn. CM with or without protease K digestion (Fig. 5A) or heat inactivation (Fig. 5B), indicating that the mutagen(s) in the Fn. CM was non-protein and heat stable. To determine the molecular weight of the mutagen(s), the Fn. CM was separated using 3-kDa MWCO membranes. Western blots and comet assay showed that < 3 kDa fractions exhibited the DNA-damaging effect compared with the control group (Fig. 5C–E). These data indicated that molecules of < 3 kDa in size that were released from *F. nucleatum* induced DNA damage in colon cells.

**Fig. 6:**
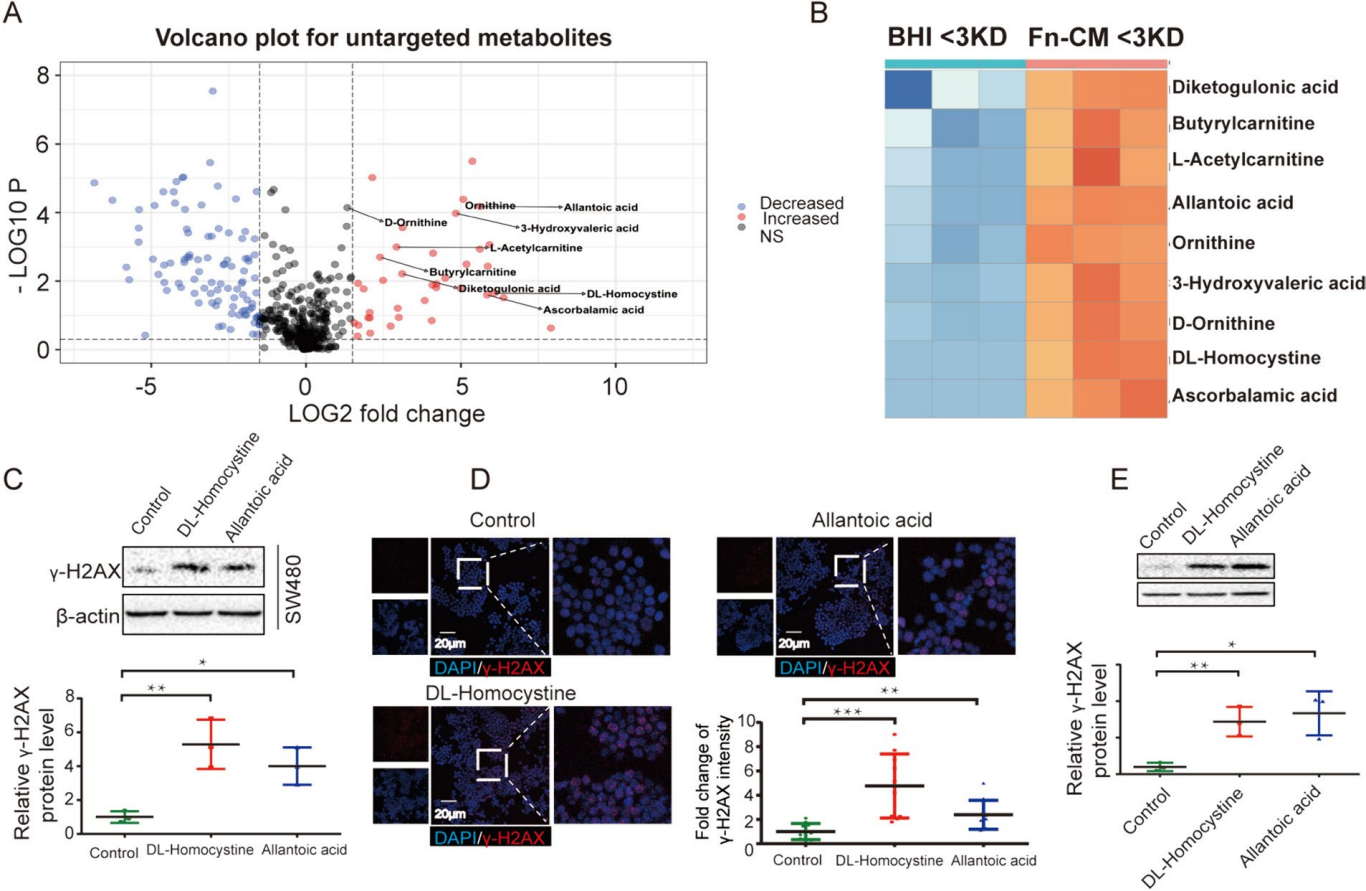
Metabolites detected from *F. nucleatum*-conditioned medium (Fn-CM) fraction which below 3KD induces DNA damage in vitro and ex vivo. Metabolites were enriched from untargeted metabolites results by volcano plot **A** and heatmap (**B**). Colon cell lines SW480 were exposed to *F. nucleatum* metabolites DL-Homocystine and Allantoic acid for 3 consecutive days. DNA damages were assayed by Western Blot (**C**) and Immunofluorescence (**D**). **E** Colon loop model was performed with PBS, DL-Homocystine or Allantoic acid. DNA damages were assayed by Western Blot. ***p ≤ 0.001; **p ≤ 0.01; *p < 0.05. Results of C-D were derived from three independent experiments

To further identify the *F. nucleatum*-secreted mutagen in < 3-kDa fractions of Fn. CM, we performed untargeted metabolomic analysis (using LC–MS/MS). A significantly higher levels of 9 metabolites, including DL-homocystine, allantoic acid, diketogulonic acid, butyrylcarnitine, L-acetylcarnitine, ornithine, 3-hydroxyvaleric acid, D-ornithine, and ascorbalamic acid were identified by volcano plot (Fig. 6A) and heatmap (Fig. 6B). Among these, DL-homocystine and allantoic acid were associated with *F. nucleatum* from KEGG pathway enrichment and were found to elevate γ-H2AX protein levels (Fig. 6C) and γ-H2AX foci number (Fig. 6D) in SW480 cells, indicating that DL-homocystine and allantoic acid might be responsible for inducing DNA damage in vitro. Importantly, in the colon loop model, DL-homocystine and allantoic acid exhibited DNA damaging property as shown by Western blots (Fig. 6E). The above results indicated that DL-homocystine and allantoic acid from < 3 kDa fractions of Fn. CM have mutagenic property.

## Discussion

According to the latest Global Cancer Statistics, colorectal cancers account for about 10% of all 19.3 million new cancer cases globally, and about 9.4% of all 9.9 million deaths [68]. The intestinal microbiota, as an important driver of health and disease, is closely associated with both the development of colorectal cancer and the efficacy of anti-tumor immunity [69, 70]. Tumorigenic bacteria can damage DNA via inducing DSBs in epithelial cells, triggering cell-cycle arrest, activation of DNA repair pathways, apoptosis, and senescence [29, 71]. DSBs is the most dangerous type of DNA damage. Although host cells have two distinct DNA DSBs repair mechanisms, namely homologous recombination (HR) and non-homologous end-joining (NHEJ), if deployed in an inappropriate cellular context, DSBs will lead to genetic instability, gross chromosomal rearrangement and accumulation of mutations, which in turn enhance cancer development if these mechanisms are impaired [72, 73]. Our TCGA cohort analysis showed that *F. nucleatum* transcriptomic abundance is positively correlated with mutation load. DNA damage response-related genes are also differentially expressed between *F. nucleatum*-high and *F. nucleatum*-low in primary CRC tissues. *F. nucleatum*-secreted molecules also exhibited genotoxic properties according to the γH2AX assay. Importantly, long-term *F. nucleatum* infection increased the number of somatic structural variation (insertion, deletion and inversion) in mouse colon tissue.

Previous studies by others support a positive role of *F. nucleatum* in CRC development and progression [11, 74]. Our study results showed that *F. nucleatum* in primary CRC tissues has a close relationship with DNA damage and poorer patients’ survival. Consistent with TCGA cohort analysis, *F. nucleatum* induced DNA damage in CRC cells. Our in vitro experiments were mainly conducted on two colon cancer cell lines, including HCT116 and SW480. It has been reported that DUOXA2, which may contribute to chronic inflammation and reactive oxygen species (ROS)-related DNA damage, was strongly upregulated in human colonic epithelial cells in response to *F. nucleatum* [75], suggesting that *F. nucleatum* not only induced DNA damage in colorectal cancer cells but also in normal colon cells. Additionally, we verified that *Fusobacterium nucleatum* induces DNA damage in the normal human colon cell line, NMC460. There is a tight relationship between DNA damage and alteration in epigenetic characteristics [76–78]. While our research did not specifically delve into the epigenetic modifications of HCT116 and SW480 cells in response to *Fusobacterium nucleatum*, it has been reported that epigenomic alterations of H3K27ac sustained in HCT116 at 24 h following initial infection with *F. nucleatum* [79]. Seldom study reported the epigenetic modifications in SW480 cells in response to *Fusobacterium nucleatum*. Future studies could benefit from incorporating an analysis of these epigenetic characteristics to provide a more comprehensive understanding of the mechanisms.

Further experiments demonstrated the DNA-damaging effect of *F. nucleatum* was mainly caused by bacteria-secreted mutagens in the Fn. CM. Metabolites, as a powerful identification tool, have been in the spotlight for cancer diagnosis, monitoring, and therapy in biological samples [80]. In our further study, the mutagenic properties of DL-homocystine and allantoic acid in the subfractions of Fn.CM were confirmed combined with the untargeted metabolites analysis. DL-homocystine is the double-bonded form of homocysteine, study showed that excessive level DL-homocystine fed could induce tibial dyschondroplasia (TD) in broiler chicks [81] and could lead to platelet adhesion and intimal hyperplasia in the hyperhomocystinemia rat carotid endarterectomy (CEA) model [82]. Studies have shown that Allantoic acid inhibited the AllR DNA binding motif to a lesser extent, which determined actinorhodin and undecylprodigiosin production, in Streptomyces coelicolor [83]. Our results suggested that DL-homocystine and allantoci acid fractions secreted by *F. nucleatum* have the mutagenic property of inducing DNA double-strand breaks. In colorectal cancer, up to 15–20% of cancers carry alterations in defective DNA damage response [84–86]. Combined with the results our TCGA data analysis, the changes of these two metabolites DL-homocysteine and allantoic acid, and their DNA damage effects exploration may be of great significance to the early diagnosis and prevention of colorectal cancer in the future and may help guide the pathological mechanisms and targeted treatment of CRC occurrence and development.

## Conclusions

In conclusion, *F. nucleatum* transcriptomic abundance is positively correlated with mutation load in primary colon adenocarcinoma samples according to the TCGA cohort. *F. nucleatum*-secreted molecules also exhibited genotoxic properties in vitro and in vivo. Furthermore, long-term *F. nucleatum* infection increased the number of somatic structural variation in colon tissue. DL-homocystine and allantoic acid may be two of the *Fusobacterium nucleatum*-secreted mutagens that cause DNA damage. Therefore, our finding delineates the genotoxicity of *F.nucleatum*-secreted mutagens, which provides a basis for further work to investigate the role of *F. nucleatum* in the pathogenicity of CRC.

## Methods

### TCGA patient samples acquisition

The gene expression and clinical data of CRC patients were obtained from the TCGA database (http://tcgadata.nci.nih.gov). CRC tissue samples from patients with gene expression data (430 patients with primary colon adenocarcinomas in TCGA cohort (TCGA-COAD cohort)) were obtained, and their clinicopathological index data were collected. High-throughput sequencing data from a larger patient cohort provided us with the opportunity to comprehensively study the tumor-specific microenvironment [87]. Kaplan–Meier plotter was used to assess the patient’s overall survival from TCGA-COAD. KEGG enrichment was used to analyze the pathway.

### Quantification of the relative abundance of *bacteria* in TCGA-COAD cohort

We obtained 560 unaligned RNA-seq data in fastq format of colon adenocarcinoma from The Cancer Genome Atlas (TCGA; http://tcgadata.nci.nih.gov) [88]. PathSeq pipeline [89] was used to detect the bacterial composition. PathSeq initially filters out human reads and low-quality reads, and then maps the remaining unmapped reads to gut-associated bacteria. Pre-built host genomes were obtained from the GATK Resource Bundle FTP server located in the /Bundle/pathseq/ directory. The microbial references used here include 1520 cultured bacterial genomes [90] and colon cancer-associated bacteria, rigorously identified through extensive and stringent statistical validation (CRC-enriched bacteria) [46, 91–93]. Bacterial reads were detected in 179 colorectal cancer tumor samples generated using single-end Illumina GA sequencing technology, while samples without bacterial detection were primarily generated by paired-end Illumina Hiseq sequencing technology, which may filter out bacterial sequences. Normalized values in the PathSeq output were used to assess the relative abundance of bacteria, taking into consideration species genome length and bacteria genome coverage. To investigate the impact of bacterial abundance on the host, we also downloaded the corresponding patient expression values and clinical information from the TCGA database. Additionally, the MSI and CMS subtype information of those patients were obtained from the Synapse platform (doi:.7303/syn2623706) (ref: https://www.ncbi.nlm.nih.gov/pmc/articles/PMC4636487/).

### F. nucleatum strains and culture conditions

*F. nucleatum* strain ATCC 25586 and *Escherichia coli* (E. coli) were purchased from Chinese Academy of Sciences (Shanghai, China)/American Type Culture Collection (ATCC, USA). *F. nucleatum* was cultured anaerobically at 37 °C for 24 h in Brain Heart Infusion (BHI; Difco™, BD, Rutherford, NJ, USA) broth medium before harvesting. E. coli strain was cultured aerobically at 37 °C for 8 h in Luria–Bertani (LB; Difco™, BD, Rutherford, NJ, USA) infusion broth. *F. nucleatum*-conditioned medium (Fn. CM) was harvested after 24 h culture. 50 μg/mL Protease K and 100 °C, 30 min heat inactivation were used to treat the Fn.CM. BHI < 3-kDa and Fn. CM < 3-kDa were separated using 3-kDa molecular weight cutoff (MWCO) membranes (BD, Rutherford, NJ, USA).

### Cell lines and culture

Human Colorectal Carcinoma Cells SW480 and HCT116 were purchased from the American Type Culture Collection (ATCC, USA). SW480 and HCT116 cells were cultured in Royal Park Memorial Institute (RPMI)-1640 (Gibco, USA) containing 10% fetal bovine serum (Gibco, USA) at 5% CO2 and 37 °C, and other cells were cultured in RPMI-1640 medium containing 10% (0.1 g/ml) fetal bovine serum (FBS, Gibco, USA). Comet assay is a useful technique in the detection of DNA damage, particularly DNA strand breaks [94]. SW480 and HCT116 were exposed to *F. nucleatum* (MOI = 50) or *E. coli* (MOI = 100) for 4 h per day under anaerobic condition for 3 consecutive days and *F. nucleatum* or Fn. CM for 3 consecutive days to assess the DNA damage. All bacteria bodies and debris in the culture supernatant were removed by centrifugation and filtration through a 0.22-µm membrane to obtain the conditioned medium (CM) that was then used to treat HCT116 and SW480 cells at the concentration of 1% (vol/vol) for 3 consecutive days.

### Determination of DNA damage

Neutral comet assay was performed according to the manufacturer’s instructions (Trevigen). The amount of DNA damage was quantified by determining the percentage of DNA in the tail and tail moment using Comet Score (TriTek) software. Graphs was generated using GraphPad Prism 5 (GraphPad Software, Inc.). Protein levels of γH2AX were detected by Western blot. For immunofluorescence staining of human colon cancer sections, slides were incubated with a primary antibody (γH2AX), followed by a secondary fluorescent antibody, and then DAPI to stain cell nuclei. Sections will be evaluated using laser scanning confocal microscopy (Olympus FV1000). The measuring of the foci of γH2AX, was determined using the ‘Colocalization Finder’ plugin in ImageJ.

### Western Blot

Total protein was isolated from cell pellets or colonic tissues and separated by SDS-PAGE. The separated proteins were then transferred onto polyvinylidene difluoride (PVDF) membranes (EMD Millipore, Billerica, MA, USA) for 1 h. The membranes were blocked with 10% non-fat milk in 0.05% Tris-based saline-Tween 20 for 1 h at room temperature. Subsequently, the membranes were incubated with primary antibodies overnight at 4 °C, including anti-β-actin (Cell signaling technology #4970) and anti-γ-H2AX antibodies (ab11174). Following this, the membranes were incubated with secondary antibodies at room temperature for 1 h. Protein band intensities were detected using the ECL Plus Western Blotting Detection Reagents (GE Healthcare).

### Animal model and tissue samples

C57BL/6 mice were purchased from The Laboratory Animal Services Center and fed in the Experimental Animal Center of the Chinese University of Hong Kong. All animals were maintained under appropriate conditions (22 ± 2 °C, 12-h light–dark cycle). All experiments performed on animals were approved by the Ethical and Institutional Animal Care and Use Committee of Prince of Wales Hospital of the Chinese University of Hong Kong. All procedures were approved by the Animal Care Committee of the Chinese University of Hong Kong and carried out in strict compliance with the relevant guidelines.

For the colon loop experiment, 6-week-old C57BL/6 male mice were used. The abdomen of each mouse was disinfected with ethanol solution immediately before surgery. Mice were anesthetized by the intraperitoneal administration of ketamine (80 mg/kg) and xylazine (10 mg/kg). A midline laparotomy was performed, and an about 3 cm-long colon loop was prepared by a double ligation of the colon. Care was taken to avoid interfering with the blood supply [95]. Colon loops were injected with 300 µl fresh *F. nucleatum* (1 × 10^9^ CFU), *F. nucleatum-*conditioned medium, BHI, BHI < 3-kDa or Fn. CM < 3-kDa, DL-Homocystine, and Allantoic acid, respectively. BHI and BHI < 3-kDa were used as controls in the experiment. The injections were performed once by the insertion of a 0.5-in., 27-gauge needle oblique to the intestinal lumen. After inoculation, the incision in the peritoneum, abdominal muscles, and skin was closed. After 6 h of incubation, colon samples were collected.

6-week-old and 180–220 g C57BL/6 male mice used in our experiments were randomly divided into two groups: experimental group and control group. All mice underwent 6 consecutive intraperitoneal injections of azoxymethane (AOM; 10 mg/kg) at 1-week intervals, followed by administration of 0.2 g/L ampicillin, neomycin, and metronidazole, and 0.1 g/L vancomycin in drinking water for 2 weeks. Mice were allowed to drink the water ad libitum for the duration of the experiments. After the last dose of antibiotics, 1 × 10^8^ colony forming units (CFU) of *F. nucleatum* were administered to the experimental group, and the same volume of phosphate-buffered saline (PBS) was given to the control group 5 days/week for 30 weeks.

### Whole genome sequencing

Whole genome sequencing was set out to explore the potential specificity of *F. nucleatum*-induced DNA damage in the mouse colon tissue from *F. nucleatum* or PBS control group. DNA of colon tissue was extracted by Genomic DNA Purification Kit (Promega) according to the manufacturer's instruction and performed whole human genomes at 30X coverage through Illumina novaseq system. The raw DNA sequencing reads were subjected to quality control where low-quality reads and short reads were removed by using Fastp. All clean data were aligned to the mouse MM10 (GRCm38) genome by using the BWA-MEM tool. Duplicated reads were marked by Sambamba [96]. Delly [97] was used to call structure variations.

### Statistical analysis

Log-rank Mantel-Cox test was used to compare Kaplan–Meier curves for patient’s overall survival. Wilcoxon rank-sum test was used to identify the differentially expressed genes between *F. nucleatum*-high and -low groups and the number of different structure variations between *F. nucleatum* samples and control samples. The 2-tailed unpaired t-test and Wilcoxon test were used to analyze the difference between control and experimental group in vitro and in vivo. Before performing other statistical procedures, the D'Agostino-Pearson normality test was applied to assess the normality of the data distribution. Measurement data are expressed as mean ± standard deviation (SD) from 3 independent experiments. P values less than 0.05 were considered statistically significant.

## Supplementary Information

Supplementary Material 1. Figure 1. Correlation between the abundance of bacteria and mutation load. Spearman’s correlation coefficient was used to assess the relationship between bacteria and mutation load. Only bacteria with a p-value<0.05 were shown in the figure.

Supplementary Material 2. Figure 2. Kaplan-Meier curves of overall survival in TCGA-COAD after excluding three patients with *F. nucleatum* abundance >10% and mutation rate >2.5. P values were determined by the Log-rank Mantel-Cox text. High *F. nucleatum* load: *F. nucleatum* relative abundance values (>50 percentile); low *F. nucleatum* RA values (<50 percentile). High mutation load: log10 number of non-synonymous mutation per sample >2: low mutation load: log10 number of non-synonymous mutation per sample <2.

Supplementary Material 3. Figure 3. Exposure to *F. nucleatum* induced host DNA DSBs. Colon cancer cell line SW480 and normal human colon epithelial cell line NCM460 were exposed to *F. nucleatum* (MOI=50) or *E. coli* (MOI= 100) or pks+ *E. coli* (MOI= 50) for 4 h per day under anaerobic conditions for 3 consecutive days. Cell line SW480 (A) and NCM460 (B) were then harvested and γ-H2AX protein level was analyzed by Western blots. pks+ *E.coli* was served as positive control.

Supplementary Material 4. Figure 4. Fold change of abundance of F. nucleatum in stool during F. nucleatum chronic experiment Relative to the control group at the 8th week. The abundance of F. nucleatum in mice stool was quantified by qPCR. ns indicates not significant. **** indicates p<0.0001.

Supplementary Material 5.

Supplementary Material 6.

## Data Availability

No datasets were generated or analysed during the current study.

## Abbreviations

*F. nucleatum*: *Fusobacterium nucleatum*
CRC: Colorectal cancer
Fn. CM: *F. nucleatum*-Conditioned medium
TLR4: Toll-like receptor 4
SBS: Single-base substitutions
*E. coli*: *Escherichia coli*
TP53: Tumor protein p53
CIN: Chromosomal instability
TCGA: The Cancer Genome Atlas
HR: Homologous recombination
NHEJ: Non-homologous end-joining
TD: Tibial dyschondroplasia
CEA: Carotid endarterectomy
COAD: Colon adenocarcinomas
CM: Conditioned medium
