# Supplementary figures and images for: Elucidating the genotoxicity of Fusobacterium nucleatum-secreted mutagens in colorectal cancer carcinogenesis
Source: Gut Pathog. 2024 Sep 27;16:50. doi: 10.1186/s13099-024-00640-w (PMC11438217; doi:10.1186/s13099-024-00640-w)

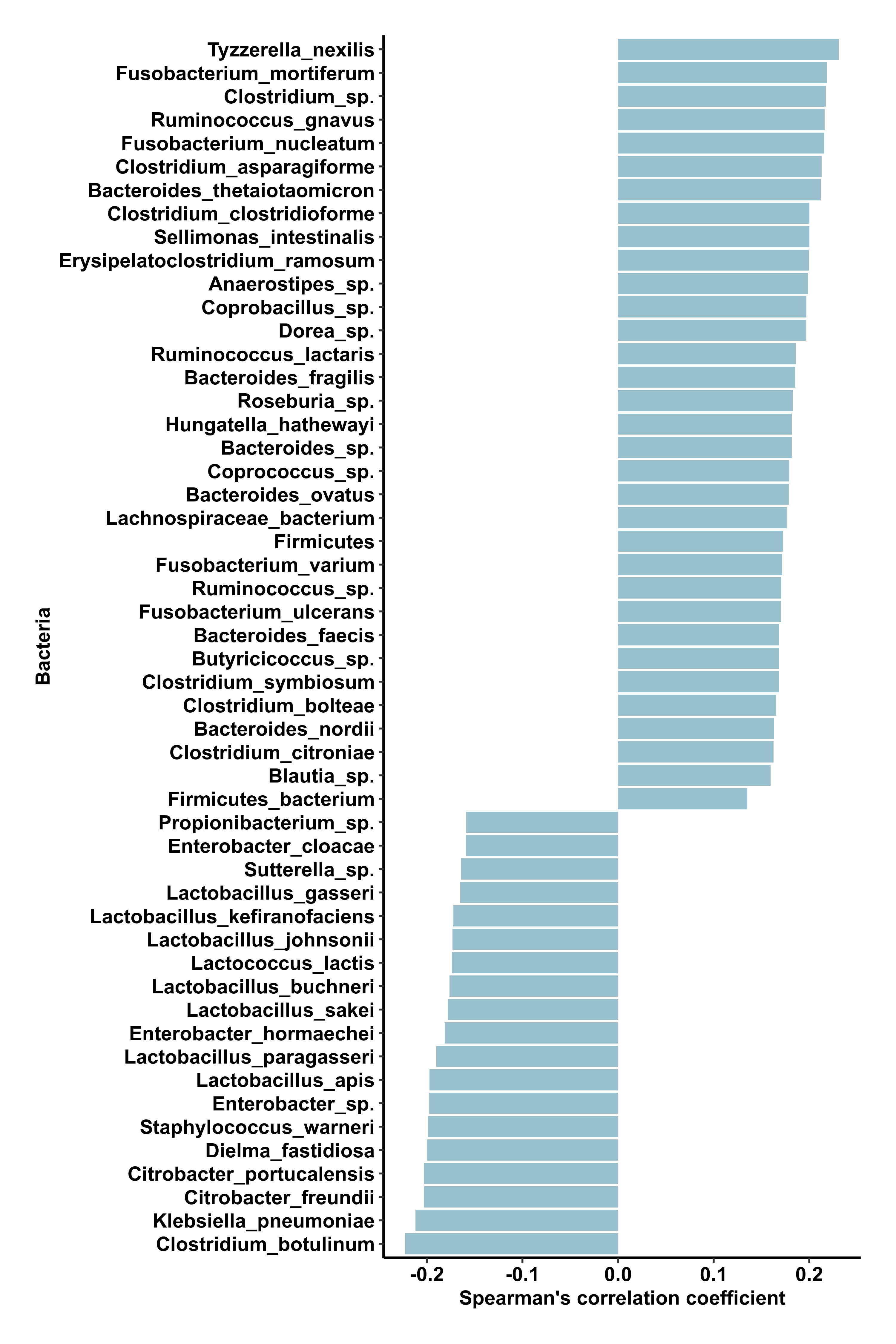

Supplement: Supplementary file 1 — Supplementary Material 1. Figure 1. Correlation between the abundance of bacteria and mutation load. Spearman’s correlation coefficient was used to assess the relationship between bacteria and mutation load. Only bacteria with a p-value<0.05 were shown in the figure. [file 13099_2024_640_MOESM1_ESM.tiff]

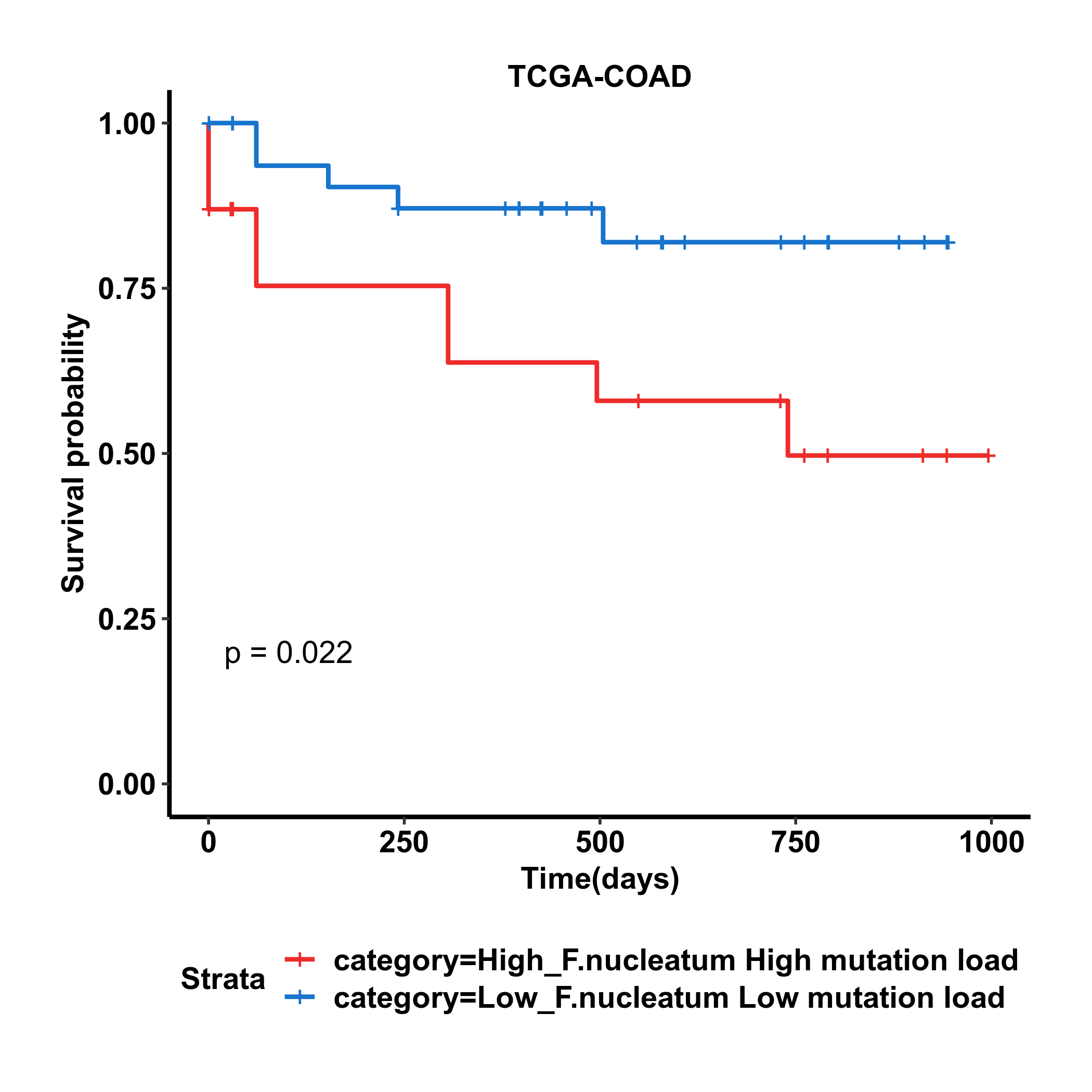

Supplement: Supplementary file 2 — Supplementary Material 2. Figure 2. Kaplan-Meier curves of overall survival in TCGA-COAD after excluding three patients with F. nucleatum abundance >10% and mutation rate >2.5. P values were determined by the Log-rank Mantel-Cox text. High F. nucleatum load: F. nucleatum relative abundance values (>50 percentile); low F. nucleatum RA values (<50 percentile). High mutation load: log10 number of non-synonymous mutation per sample >2: low mutation load: log10 number of non-synonymous mutation per sample <2. [file 13099_2024_640_MOESM2_ESM.tiff]

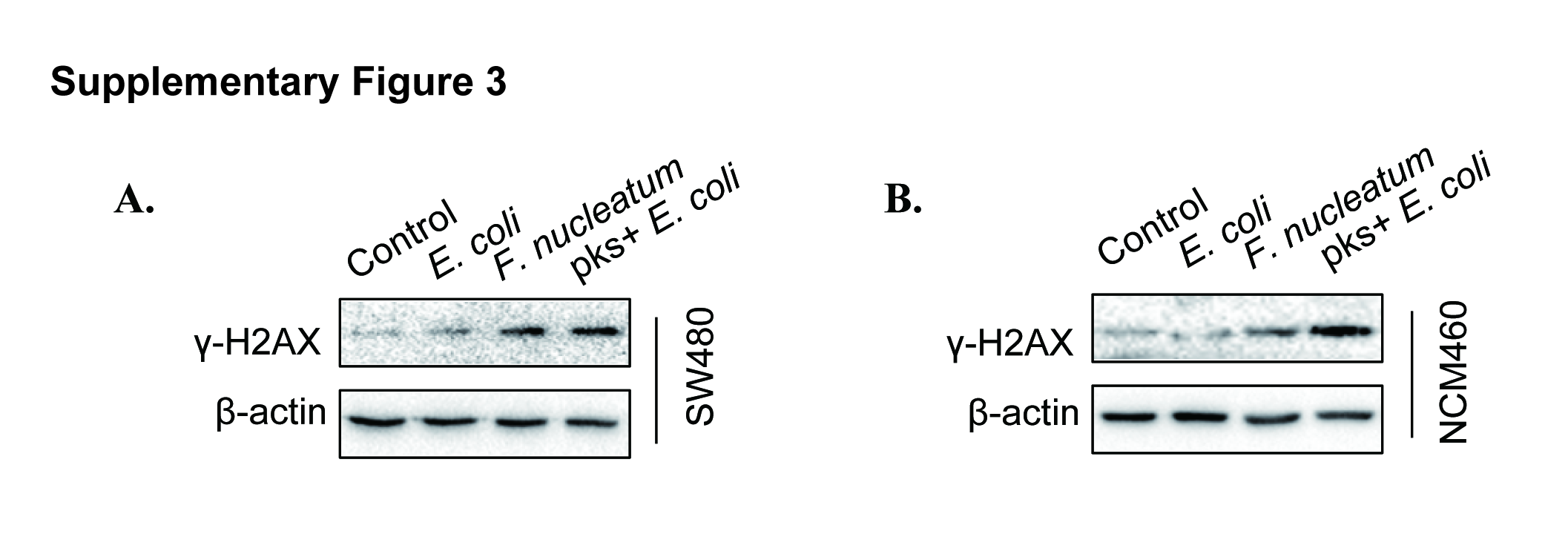

Supplement: Supplementary file 3 — Supplementary Material 3. Figure 3. Exposure to F. nucleatum induced host DNA DSBs. Colon cancer cell line SW480 and normal human colon epithelial cell line NCM460 were exposed to F. nucleatum (MOI=50) or E. coli (MOI= 100) or pks+ E. coli (MOI= 50) for 4 h per day under anaerobic conditions for 3 consecutive days. Cell line SW480 (A) and NCM460 (B) were then harvested and γ-H2AX protein level was analyzed by Western blots. pks+ E.coli was served as positive control. [file 13099_2024_640_MOESM3_ESM.tif]

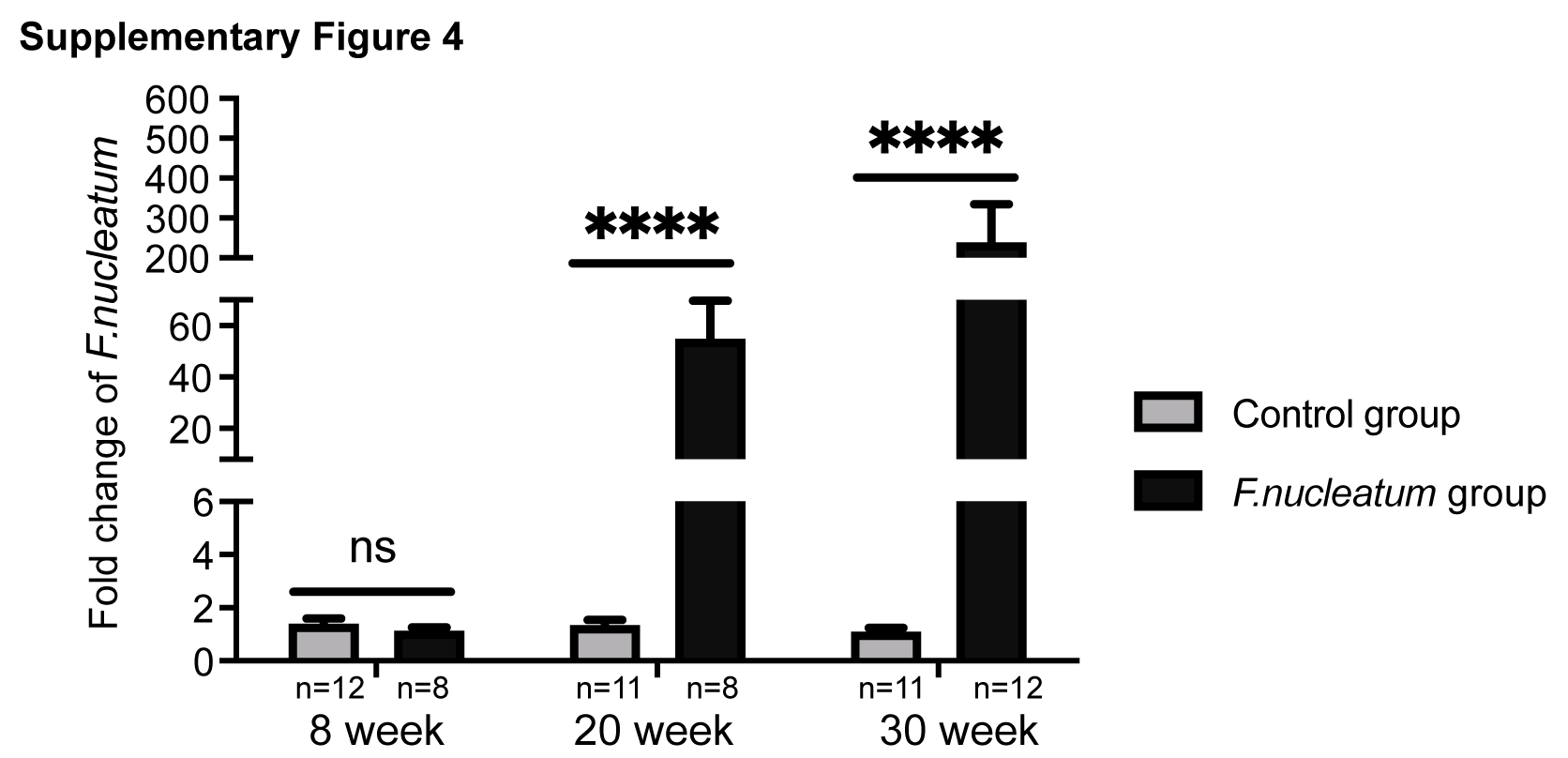

Supplement: Supplementary file 4 — Supplementary Material 4. Figure 4. Fold change of abundance of F. nucleatum in stool during F. nucleatum chronic experiment Relative to the control group at the 8th week. The abundance of F. nucleatum in mice stool was quantified by qPCR. ns indicates not significant. **** indicates p<0.0001. [file 13099_2024_640_MOESM4_ESM.tif]
