# Supplementary material for: Elucidating the genotoxicity of Fusobacterium nucleatum-secreted mutagens in colorectal cancer carcinogenesis
Source: Gut Pathog. 2024 Sep 27;16:50. doi: 10.1186/s13099-024-00640-w (PMC11438217; doi:10.1186/s13099-024-00640-w)
Supplement: Supplementary file 5 — Supplementary Material 5. [file 13099_2024_640_MOESM5_ESM.docx]

|  | TCGA-AA-3510 | TCGA-AA-3715 | TCGA-AA-3966 |
| --- | --- | --- | --- |
| Gender | male | male | female |
| Age | 70 | 77 | 89 |
| Tumor_stage.diagnoses | stage ii | stage ii | stage iia |
| MSI | NA | msi | msi |
| CMS | NA | CMS1 | CMS1 |
| kras_ mutation | NA | NA | NA |
| BRAF mutation | NA | NA | NA |
| vital_status.demographic | Alive | Dead | Alive |
| Site_of_resection_or_biopsy.diagnoses | Colon, NOS | Ascending colon | Colon, NOS |
| Disease_type | Adenomas and Adenocarcinomas | Complex Epithelial Neoplasms | Cystic, Mucinous and Serous Neoplasms |

**Supplementary Table 1. Characteristics of the patients with *F. nucleatum* abundance >10% and mutation rate >2.5**
