# Supplementary material for: Elucidating the genotoxicity of Fusobacterium nucleatum-secreted mutagens in colorectal cancer carcinogenesis
Source: Gut Pathog. 2024 Sep 27;16:50. doi: 10.1186/s13099-024-00640-w (PMC11438217; doi:10.1186/s13099-024-00640-w)
Supplement: Supplementary file 6 — Supplementary Material 6. [file 13099_2024_640_MOESM6_ESM.docx]

**Supplementary Table 2. Characteristics of colorectal cancer patients with high or low *F. nucleatum* relative abundance**

|  |  | High_F.nucleatum,n(%)* | Low_F.nucleatum,n(%)* | Total_n* | P_Value |
| --- | --- | --- | --- | --- | --- |
| Age | **Age(mean)** | 70.944 | 68.422 |  | 0.140 |
| Gender | **male** | 37(41.6) | 51(56.7) | 88 | 0.052 |
|  | **female** | 52(58.4) | 39(43.3) | 91 |  |
| Stage | **4** | 12(13.6) | 17(18.9) | 29 | 0.336 |
|  | **3** | 25(28.4) | 23(25.6) | 48 |  |
|  | **2** | 39(44.3) | 31(34.4) | 70 |  |
|  | **1** | 12(13.6) | 19(21.1) | 31 |  |
| MSI | **mss** | 64(71.9) | 83(92.2) | 147 | **3.99E-04** |
|  | **msi** | 25(28.1) | 7(7.8) | 32 |  |
| CIMP | **CIMP.Neg** | 42(50) | 62(69.7) | 104 | **2.28E-02** |
|  | **CIMP.High** | 21(25) | 11(12.4) | 32 |  |
|  | **CIMP.Low** | 21(25) | 16(18) | 37 |  |
| KRAS mutation | **No** | 21(80.8) | 28(84.8) | 49 | 0.736 |
|  | **Yes** | 5(19.2) | 5(15.2) | 10 |  |
| BRAF mutation | **No** | 24(92.3) | 33(100) | 57 | 0.190 |
|  | **Yes** | 2(7.7) | 0(0) | 2 |  |
| CMS label | **CMS4** | 13(14.6) | 20(22.2) | 33 | **5.91E-04** |
|  | **CMS1** | 27(30.3) | 6(6.7) | 33 |  |
|  | **CMS2** | 27(30.3) | 43(47.8) | 70 |  |
|  | **NOLBL** | 12(13.5) | 13(14.4) | 25 |  |
|  | **CMS3** | 10(11.2) | 8(8.9) | 18 |  |

* *P*-values were calculated using the Fisher test, except for age where the Wilcoxon test was utilized.
